# *Streptococcus zooepidemicus* Meningitis in an HIV-Positive Horse Breeder Patient: A Case Study and Literature Review

**DOI:** 10.3390/idr15050052

**Published:** 2023-09-07

**Authors:** Petya Argirova, Yordan Kalchev, Ivan Baltadzhiev, Mariyana Stoycheva, Marianna Murdjeva

**Affiliations:** 1 Department of Infectious Diseases, Parasitology and Tropical Medicine, Faculty of Medicine, Medical University-Plovdiv, 4002 Plovdiv, Bulgaria; ivan.baltadzhiev@mu-plovdiv.bg; 2 Department of Medical Microbiology and Immunology “Prof. Dr. ElissayYanev”, Faculty of Pharmacy, Medical University-Plovdiv, 4002 Plovdiv, Bulgaria; yordan.kalchev@mu-plovdiv.bg (Y.K.); mmurdjeva@yahoo.com (M.M.); 3 Laboratory of Microbiology, St. George University Hospital, 4002 Plovdiv, Bulgaria; 4 Research Institute, Medical University-Plovdiv, 4002 Plovdiv, Bulgaria; marianavartigova49@gmail.com; 5 Clinic for Infectious Diseases, St. George University Hospital, 4001 Plovdiv, Bulgaria

**Keywords:** meningitis, neuroinfections, *Streptococcus zooepidemicus*, *Streptococcus equi*, horses, HIV, AIDS, group C streptococci, streptococcal infections, central nervous system

## Abstract

*Streptococcus equi* subsp. *zooepidemicus* is a rare etiologic agent of bacterial meningitis in humans. The disease is a zoonotic infection and is transmitted through close contact with domestic animals, mainly horses. Only 37 cases of *Streptococcus zooepidemicus* meningitis have been reported in the literature until July 2023. The aim of this study is to present a rare clinical case of *S. zooepidemicus*-related meningitis in a human immunodeficiency virus (HIV)-positive patient and analyze the literature. We present a 23-year-old horse breeder patient with advanced immunosuppression due to acquired immunodeficiency syndrome (AIDS) and *S. zooepidemicus* meningitis, admitted at the Clinic of Infectious Diseases, St. George University Hospital, Plovdiv. The course of meningitis was severe since the beginning, with significant cerebral edema, disturbances in consciousness, persistent fever, and the development of complications against the background of AIDS-related conditions. *S. zooepidemicus* was microbiologically detected from cerebrospinal fluid culture. After prolonged treatment and a long hospital stay, the patient’s condition improved, and eventually he was discharged and recovered from the acute neuroinfection. Although extremely rare, *S. zooepidemicus* should be considered in patients with clinical and laboratory evidence of bacterial meningitis who have contact with animals, especially horses, other domestic animals, and their dairy products, as well as in immunocompromised patients. To the best of our knowledge, the current clinical case is the first report of *S. zooepidemicus*-related meningitis in a patient with HIV/AIDS.

## 1. Introduction

*Streptococcus equi* subsp. *zooepidemicus (S. zooepidemicus)* belongs to a group of β-hemolytic streptococci with Lancefield group C antigen, along with *S. dysgalactiae* subsp. *dysgalactiae*, *S. dysgalactiae* subsp. *equisimilis*, *S. equi* subsp. *equi*. Group C streptococci are not considered part of the normal human flora. They are recognized as either commensals or pathogens in a wide array of domestic animals and are uncommon causes of infections in humans [1].

*S. zooepidemicus* is a commensal in the skin and mucous membranes of the upper respiratory tract of horses but can cause rhinitis, bronchitis, pneumonia, arthritis, submandibular lymphadenitis, and wound infection as an opportunistic pathogen [2]. In humans, it causes uncommon zoonotic diseases with very few reported cases. Infections range from mild diseases such as pharyngitis and skin and soft tissue infections to a severe clinical presentation of epiglottitis, pneumonitis, septic arthritis, osteomyelitis, peritonitis, sepsis, endocarditis, and meningitis [3].

*S. zooepidemicus* is a rare etiologic agent of bacterial meningitis in humans. Nearly all of the meningitis cases have been attributed to zoonotic exposure. Horses are implicated in most cases of direct animal contact. Other domestic animals like dogs, swine, cattle, guinea pigs, and sheep can also serve as sources of infections in humans. Whenever possible, the suspect animal is tested for *S. zooepidemicus* from nasal or nasopharyngeal secretions [1]. Another transmission mechanism is through the consumption of unpasteurized milk and dairy products. The incubation period varies from 1 to 21 days (median 7 days). Benzylpenicillin and third-generation cephalosporins are the antibiotics of choice for group C streptococcal infections. Gentamicin or rifampin can be added for a synergistic effect against this pathogen [3,4].

## 2. Case Presentation

A 23-year-old male was admitted to the Clinic of Infectious Diseases at the St. George University Hospital–Plovdiv in Bulgaria with fever, vomiting, diarrhea, rhinitis, sore throat, and cough over the last 2 days. According to his relatives, he had episodes of nosebleeding and bloody urine. On the day of admission, he became aggressive and confused, and soon after, he progressed to a comatose state. Relevant comorbidities included Human Immunodeficiency Virus (HIV) infection stage C—Acquired Immunodeficiency Syndrome (AIDS), chronic hepatitis B, hepatitis C, liver cirrhosis, esophageal varices, and wasting syndrome. The infection with HIV was established 7 years ago when antiretroviral therapy was initiated. The therapy included Combivir (lamivudine, zidovudine) and Kaletra (lopinavir, ritonavir). A serious problem in our patient was his non-adherence to antiretroviral therapy (ART), with frequent and long-term interruptions in medication intake, which resulted in very poor control of HIV infection. Also, he had not taken ART for about a year before the onset of meningitis. In addition, he was an intravenous drug user and had also recovered from a middle ear infection (otitis media) 2 months before. On physical examination, the patient was febrile (39 °C), dehydrated, comatose, had nuchal stiffness, and had a positive Babinski sign. He had tachycardia with a heart rate of 120 beats per minute and hypotension of 100/60 mmHg. On auscultation of the chest, crackles were heard over both lungs. Splenomegaly, catarrhal angina, skin petechiae, and trophic changes on the legs were also present.

Results of routine laboratory tests showed anemia with hemoglobin of 90 g/L (reference for males 140–180 g/L), leukocytosis with white blood cell count (WBC) of 15.8 × 10^9^/L (reference 3.5–10.5 × 10^9^/L), followed by severe leukocytopenia (WBC varied from 1.4 to 2.8 × 10^9^/L), thrombocytopenia with platelets varying from 31 to 60 × 10^9^/L (reference 140–440 × 10^9^/L), elevated C-reactive protein of 79 mg/L (reference 0–10 mg/L), an erythrocyte sedimentation rate of 44 mm/h (reference for males below the age of 50 < 15 mm/h), and a low sodium level of 126 mmol/L (reference 136–151 mmol/L). An emergency computed tomography (CT) scan of the head revealed no abnormalities. A lumbar puncture (LP) was performed immediately. The cerebrospinal fluid (CSF) analysis for the whole hospital stay is presented in Table 1. The microscopic evaluation showed blood contamination of the CSF sample obtained from the second LP.

Unfortunately, technical difficulties occurred during the second lumbar puncture. It is likely that a small blood vessel was accidentally ruptured, causing unwanted blood contamination of the cerebrospinal fluid.

Empiric antibiotic therapy was initiated with cefotaxime 4 × 2 g i.v., vancomycin 3 × 1 g i.v., and trimethoprim/sulfamethoxazole 2 × 960 mg i.v. Furthermore, as an adjunctive treatment, dexamethasone and mannitol 10% were administered.

Gram and methylene blue staining of the CSF specimen revealed no inflammatory cells or microorganisms. An overnight incubation on 5% sheep blood agar revealed white colonies with β-hemolysis that tested catalase-negative. The bacterium was also recovered from blood cultures. Both strains were identified as *S. equi* subsp. *zooepidemicus* by Vitek-2 Compact (bioMerieux, France). The strain was susceptible to benzylpenicillin, cefotaxime, cefepime, erythromycin, teicoplanin, and linezolid but resistant to clindamycin. A diagnosis of *S. zooepidemicus* meningitis was made. Because of the patient’s HIV-positive status, additional tests were performed. Sputum cultures were positive for *Acinetobacter baumannii*, and stool cultures were negative. Serology showed negative IgM antibodies to the Ebstein-Barr virus and Cytomegalovirus but positive IgG antibodies for both viruses. The patient tested positive for hepatitis B surface antigen and also for hepatitis C virus antigens and antibodies. Parasitology tests were negative for toxoplasmosis and pneumocystosis (Giemsa stain). HIV viral load was 2785 copies/mL and CD4+ T-cells were only 35/mm^3^.

**Figure 1:**
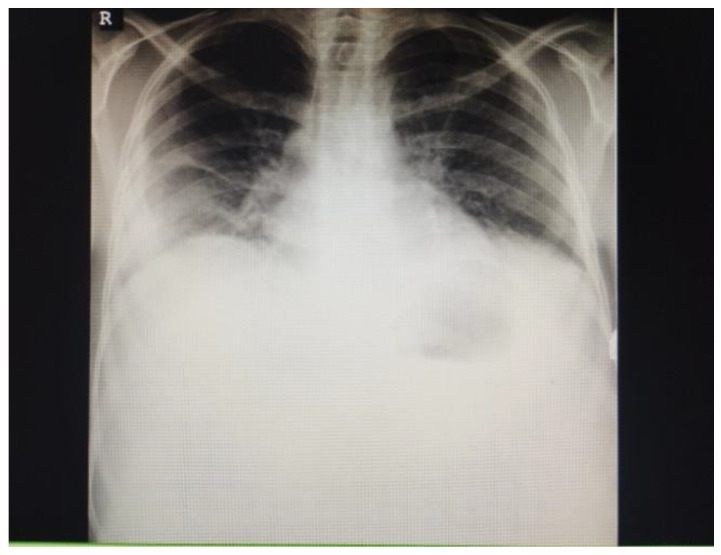
Chest radiograph. Right axillary and basal inflammatory infiltrates. R-right side.

**Figure 2:**
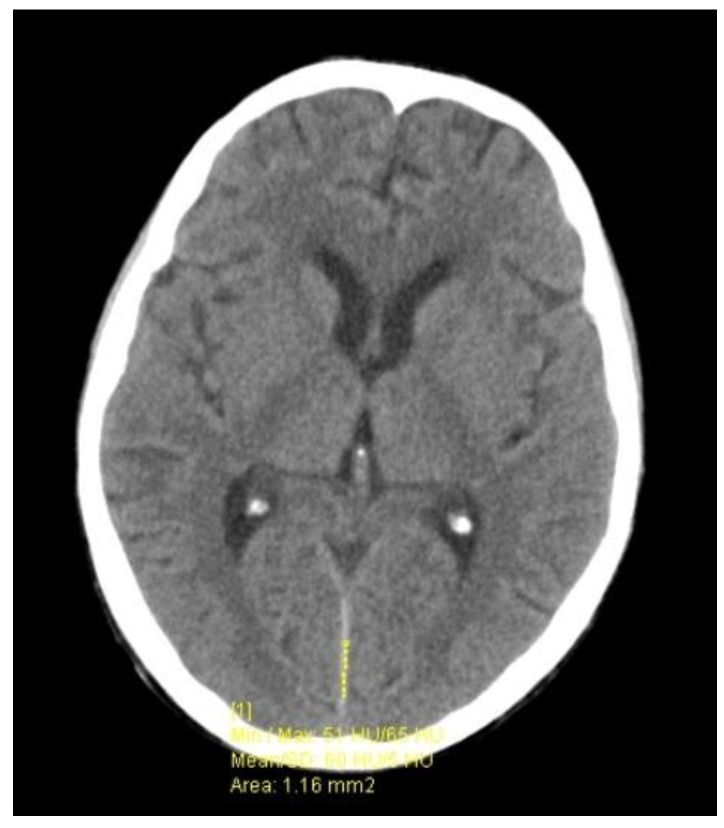
Control head CT. No parenchymal lesions. Tentorium and falx cerebelli appeared slightly thickened with a density of 61 Hounsfield units (suspicion of a small subarachnoid hemorrhage).

Regardless of the initiated treatment, the patient continued to be somnolent and confused; fever persisted at over 38.5 °C for 15 days; and anemia worsened over the first 7 days of admission. Skin and mucous hemorrhages, hematuria, jaundice, and generalized edema appeared as a result of his decreased platelet numbers as well as chronic liver failure. The latter was supported by severe laboratory abnormalities such as increased aspartate transaminase of 157 U/L (reference 0–36 U/L), alanine transaminase of 69 U/L (reference for males 0–49 U/L), total bilirubin of 60 µmol/L (reference 3.4–21 µmol/L), decreased serum cholinesterase of 1180 U/L (reference 2100–5000 U/L), albumin 21 g/L (reference 35–55 g/L), prothrombin time varied from 16.6 to 51.7% (reference 70–120%), fibrinogen to 1.18 g/L (reference 2–4.5 g/L). Three weeks after the admission, chest radiography revealed pneumonia (Figure 1), and an abdominal ultrasound showed signs of liver cirrhosis. A second control CT of the brain was performed, which revealed suspicion of a small subarachnoid hemorrhage (Figure 2).

Due to the lack of clinical improvement and the persistence of fever, bacteremia, and pneumonia, the therapy was changed to benzylpenicillin 6 × 4,000,000 IU i.v. and teicoplanin 2 × 0.4 g i.v. The combination of cefotaxime and vancomycin was administered for an overall period of 14 days. After starting the new antimicrobial therapy, the patient’s condition slowly began to improve. Benzylpenicillin was continued for 14 days and teicoplanin for 21 days. During acute bacterial meningitis, the permeability of the blood–brain barrier is increased, so we could speculate that teicoplanin could have reached sufficient concentrations within the subarachnoid space. Massive amounts of erythrocyte and platelet concentrates, fresh-frozen plasma, human albumin 20%, and symptomatic drugs were needed until liver functions stabilized. The patient recovered after a hospital stay of 41 days. He was eventually discharged with no neurological sequelae.

## 3. Discussion

By reviewing the medical literature, we were able to find 32 cases of meningitis due to *S. zooepidemicus* until April 2022 (Table 2) [5,6,7,8,9,10,11,12,13,14,15,16,17,18,19,20,21,22,23,24,25,26,27,28,29,30]. *S. pneumoniae*, *N. meningitidis*, *L. monocytogenes*, and *Staphylococcus* spp. are common etiologic agents of acute bacterial meningitis in humans, whereas group C streptococci can rarely cause inflammation of the meninges [31]. Recently, Bosica S et al. published a study about the *S. zooepidemicus* outbreak associated with the consumption of unpasteurized dairy products in Italy. It involved 37 people who were infected, of whom 35 were symptomatic. A wide range of clinical manifestations were observed, including septicemia, pharyngitis, arthritis, uveitis, and endocarditis. Five of the patients developed severe meningitis and subsequently died [32]. Unfortunately, there were no more clinical details about the reported meningitis cases in this study.

Meningitis caused by *S. zooepidemicus* presents with clinical and laboratory findings characteristic of purulent meningitis [3]. However, the CSF parameters from the initial LP in our patient were within normal limits, which can be explained by the severe immunosuppression due to AIDS, and the inflammatory response within the subarachnoid space is not manifested so vividly in the course of the disease. We consider that the marked increase in CSF WBC of 6826 × 10^6^/L can be explained by the blood contamination of the second CSF sample.

Epidemiological data have a valuable influence on the diagnosis of this zoonotic disease. We know the source of *S. zooepidemicus* in our patient. The patient had daily close contact with his horses while taking care of them, so we could establish the most probable source for the infection.

According to the reported cases in the literature (n = 32), the average age of the patients was 49.8 years, ranging from 1 day to 83 years. The majority (84%) were adults (over 18 years old), with an equal gender distribution (1:1). An exposure to the pathogen was reported in 90.4% of the patients, such as contact with horses (53%), consumption of unpasteurized cow or goat milk and milk products in 28%, and contact with symptomatic dogs (6%). Comorbidities were present in 52%, most often cardiovascular diseases (33.3%), arterial hypertension (14.8%), and diabetes mellitus (7.4%).

To the best of our knowledge, there were no other reported patients with HIV/AIDS or *S. zooepidemicus* meningitis. Similarly to our patient, concomitant bacteremia (68%) and pneumonia (13%) were established by other authors. Furthermore, otogenic disorders such as otitis, sinusitis, mastoiditis (16%), endocarditis (10%), and endophthalmitis (7%) were observed in these reports. An interesting fact is that 68% of patients have microbiologically proven bacteremia, although only a few cases meet the clinical and laboratory criteria for sepsis [5,18]. The most commonly reported antibiotics for *S. zooepidemicus* meningitis were 3^rd^ generation cephalosporins (ceftriaxone, cefotaxime), benzylpenicillin, ampicillin, and vancomycin. Gentamycin and rifampicin were rarely administered in the reported cases. Currently, there are no guidelines for the antimicrobial treatment of meningitis in HIV patients caused by *S. zooepidemicus*. We consider the duration of the antimicrobial treatment to be determined by the clinical course and laboratory parameters. The lethality rate was 22.6%. Residual neurological sequelae such as deafness (16.1%) and visual disturbances (9.7%) were registered in the survivors.

## 4. Conclusions

In summary, the presented case report confirms the role of *S. zooepidemicus* as a possible zoonotic pathogen in patients with acute bacterial meningitis. Although extremely rare, *S. zooepidemicus* should be considered in patients with clinical and laboratory evidence of bacterial meningitis who have had contact with animals, especially horses, or consumed unpasteurized milk. In addition, this case of *S. zooepidemicus* meningitis in a patient with HIV infection is the first reported in the literature, and it may extend our knowledge on the role of this pathogen in immunocompromised patients. With the current case report, we will update the epidemiological data on the etiology of bacterial meningitis in HIV individuals.

## Figures and Tables

**Table 1:** CSF analysis on admission (on day 1) and control LPs (on days 8 and 16).

Parameters	1st LP (Day 1)	2nd LP (Day 8)	3rd LP (Day 16)
WBC, ×10^6^/L	8	6826	271
Protein, g/L	0.19	1.22	0.55
Glucose, mmol/L	1.50	1.30	3.4
Serum glucose, mmol/L	1.52	5.2	5.6
Ratio *	0.99	0.25	0.61

CSF—cerebrospinal fluid; WBC—white blood cells; LP—lumbar puncture. * CSF glucose/serum glucose ≤ 0.4 is suspicious for bacterial meningitis.

**Table 2:** Cases of human meningitis caused by *S. zooepidemicus* reported until April 2023 (n = 32).

Age	Sex	Concomitant Conditions	Exposure	Comorbidities	Outcome	Ref.
1 day	M	Bacteremia, pneumonia	Unpasteurized milk	Prematurity	Died	[5]
3 mo	M	Bacteremia	Unknown	nd	Recovered	[6]
5 mo	M	Bacteremia, cerebral infarction	Unknown	None	Deafness, quadriplegia	[7]
6 mo	F	Bacteremia	Ill dogs	None	Deafness	[8]
13 yr	F	Bacteremia	Horse	Asthma/allergy	Deafness	[9]
24 yr	M	None	Horses	None	Recovered	[10]
24 yr	F	Bacteremia	Horse	None	Recovered	[11]
30 yr	F	nd	Horses	Cocaine use	Recovered	[12]
33 yr	M	Bacteremia	Unknown	nd	Deafness	[13]
37 yr	F	Brain abscesses	Horse bite	None	Hemiparesis, aphasia	[14]
41 yr	M	Bacteremia, sinusitis	Horse manure	None	Recovered	[15]
41 yr	F	Cardiac and respiratory failure	Horses	None	Died	[14]
49 yr	F	Bacteremia	Horse	None	Diplopia	[16]
51 yr	F	Bacteremia, sinusitis, mastoiditis	Horse	Diabetes	Recovered	[4]
56 yr	F	None	Horses	Hepatitis C	Recovered	[17]
57 yr	M	Bacteremia, sepsis, endocarditis	Horses	AoVI	Prolonged recovery	[18]
59 yr	F	Bacteremia	Unpasteurized milk	None	Slow recovery	[19]
59 yr	F	Bacteremia, endophtalmitis, endocarditis	Sporadic contact with horses	Polymorbid *	Blindness, deafness	[20]
59 yr	M	Otitis, mastoiditis, pneumonia	Unpasteurized cheese	Hypertension, stroke	Recovered	[21]
66 yr	M	Bacteremia	Ill dogs	None	Recovered	[22]
67 yr	F	Otitis	Cattle farmer	None	Recovered	[23]
71 yr	M	Bacteremia	Unpasteurized milk	VT	Recovered	[5]
72 yr	F	None	Horse	MI	Prolonged recovery	[24]
73 yr	M	Bacteremia	Unpasteurized milk	nd	Died	[5]
73 yr	M	Bacteremia, endocarditis	Unpasteurized milk	Aortic aneurysm	Died	[5]
73 yr	M	Bacteremia, endophtalmitis, pneumonia	Horse	MI	Impaired visual acuity	[25]
73 yr	F	Otitis	Unpasteurized milk	Polymorbid **	Recovered	[26]
74 yr	M	Bacteremia	Horses	None	Recovered	[27]
75 yr	M	Bacteremia	Horse	None	Died	[28]
79 yr	M	Wound infection	Horses	nd	nd	[29]
80 yr	F	Bacteremia	Unpasteurized milk	nd	Died	[5]
83 yr	F	Pneumonia	Unpasteurized cheese	Hypertension	Died	[30]

mo—months; yr—years; M—male; F—female; nd—no data; VT—venous thrombosis; MI—myocardial infarction; AoVI—aortic valve; insufficiency; * arterial hypertension, diabetes mellitus, myocardial infarction, chronic renal failure, hypothyroidism; ** arterial hypertension, obesity, chronic obstructive pulmonary disease, osteodural defect.

## Data Availability

Data are not available.

## References

[1] Baracco G.J. (2019). Infections Caused by Group C and G Streptococcus (*Streptococcus dysgalactiae* subsp. equisimilis and Others): Epidemiological and Clinical Aspects. Microbiol. Spectr..

[2] Facklam R. (2002). What happened to the streptococci: Overview of taxonomic and nomenclature changes. Clin. Microbiol. Rev..

[3] Trell K., Nilson B., Petersson A.C., Rasmussen M. (2017). Clinical and microbiological features of bacteremia with *Streptococcus equi*. Diagn. Microbiol. Infect. Dis..

[4] Minces L.R., Brown P.J., Veldkamp P.J. (2011). Human meningitis from *Streptococcus equi* subsp. zooepidemicus acquired as zoonoses. Epidemiol. Infect..

[5] Edwards A.T., Roulson M., Ironside M.J. (1988). A milk-borne outbreak of serious infection due to Streptococcus *zooepidemicus* (Lancefield Group C). Epidemiol. Infect..

[6] Jenkins E.L., McGuire W. (2000). Group C streptococcal meningitis in infancy. Acta Paediatr..

[7] Sevilla-Acosta F., Ballestero-Pernudi A., Jiménez-Cruz E., Álvarez-Cabalceta H., Naranjo-Zuñiga G. (2021). *Streptococcus equi* subspecies *zooepidemicus* Meningitis, Septicemia, and Brain Infarcts in a Costa Rican Infant. Cureus.

[8] Zahlanie Y., Almatrafi M., Filkins L., Hsiang M.S. (2019). Possible canine source of *Streptococcus equi* subspecies *zooepidemicus* causing meningitis in an infant. IDCases.

[9] Shah S.S., Matthews R.P., Cohen C. (2001). Group C streptococcal meningitis: Case report and review of the literature. Pediatr. Infect. Dis. J..

[10] Cheeseman M., Genain C., Smith C.D. (1990). Group C streptococcal meningitis with favorable recovery. A case report. J. Ky. Med. Assoc..

[11] Low D.E., Young M.R., Harding G.K. (1980). Group C streptococcal meningitis in an adult. Probable Acquistion A Horse. Arch. Intern. Med..

[12] Rivas M.T., Pascual J., Sesar A. (2008). Group C Streptococcus meningitis: A very uncommon condition. Neurologia.

[13] Patey O., Buisson C.B., Soussy C.J. (1990). Group C streptococcal meningitis in adults. Rev. Infect. Dis..

[14] van Samkar A., Brouwer M.C., van der Ende A., van de Beek D. (2016). *Streptococcus equi* meningitis. Clin. Microbiol. Infect..

[15] Pati S., Al-Araji A., Orendi J. (2007). Atypical presentation of *Streptococcus zooepidemicus* bacteraemia and secondary meningitis. Clin. Neurol. Neurosurg..

[16] Downar J., Willey B.M., Sutherland J.W., Mathew K., Low D.E. (2001). Streptococcal meningitis resulting from contact with an infected horse. J. Clin. Microbiol..

[17] Klapa S., Grefer J., Sobottka I., Kurowski V. (2021). A 56-Year-Old Woman with Chronic Hepatitis C Liver Disease and Meningitis due to *Streptococcus equi* subsp. Zooepidemicus. Case Rep. Crit. Care.

[18] Pelkonen S., Lindahl S.B., Suomala P., Karhukorpi J., Vuorinen S., Koivula I., Väisänen T., Pentikäinen J., Autio T., Tuuminen T. (2013). Transmission of *Streptococcus equi* subspecies *zooepidemicus* infection from horses to humans. Emerg. Infect. Dis..

[19] Mohr D.N., Feist D.J., Washington J.A., Hermans P.E. (1978). Meningitis due to group C streptococci in an adult. Mayo Clin. Proc..

[20] Poulin M.F., Boivin G. (2009). A case of disseminated infection caused by *Streptococcus equi* subspecies *zooepidemicus*. Can. J. Infect. Dis. Med. Microbiol..

[21] Mori N., Guevara J.M., Tilley D.H. (2013). *Streptococcus equi* subsp. zooepidemicus meningitis in Peru. J. Med. Microbiol..

[22] Ghoneim A.T., Cooke E.M. (1980). Serious infection caused by group C streptococci. J. Clin. Pathol..

[23] Latorre M., Alvarez M., Fernández J.M., Berdonces P., Llanos A., Cisterna R. (1993). A case of meningitis due to “Streptococcus *zooepidemicus*”. Clin. Infect. Dis..

[24] Jovanović M., Stevanović G., Tošić T., Stošović B., Zervos M.J. (2008). *Streptococcus equi* subsp. zooepidemicus meningitis. J. Med. Microbiol..

[25] Madžar D., Hagge M., Möller S., Regensburger M., Lee D.H., Schwab S., Jantsch J. (2015). Endogenous endophthalmitis complicating *Streptococcus equi* subspecies *zooepidemicus* meningitis: A case report. BMC Res. Notes.

[26] Aida Z., Lamia A., Souheil Z., Badreddine K., Monika B., Rim A., Aida B., Hajer H., Hanen T.B. (2020). Meningitis due to *Streptococcus equi* in a 73 year old woman with an osteodural defect. ID Cases.

[27] Ferrandière M., Cattier B., Dequin P.F., Hazouard E., Legras A., Perrotin D. (1998). Septicemia and meningitis due to Streptococcus *zooepidemicus*. Eur. J. Clin. Microbiol. Infect. Dis..

[28] Ural O., Tuncer I., Dikici N., Aridogan B. (2003). Streptococcus *zooepidemicus* meningitis and bacteraemia. Scand. J. Infect. Dis..

[29] Eyre D.W., Kenkre J.S., Bowler I.C., McBride S.J. (2010). *Streptococcus equi* subspecies *zooepidemicus* meningitis--a case report and review of the literature. Eur. J. Clin. Microbiol. Infect. Dis..

[30] Bordes-Benítez A., Sánchez-Oñoro M., Suárez-Bordón P., García-Rojas A.J., Saéz-Nieto J.A., González-García A., Alamo-Antúnez I., Sánchez-Maroto A., Bolaños-Rivero M. (2006). Outbreak of *Streptococcus equi* subsp. zooepidemicus infections on the island of Gran Canaria associated with the consumption of inadequately pasteurized cheese. Eur. J. Clin. Microbiol. Infect. Dis..

[31] van de Beek D., Cabellos C., Dzupova O., Esposito S., Klein M., Kloek A.T., Leib S.L., Mourvillier B., Ostergaard C., Pagliano P. (2016). ESCMID Study Group for Infections of the Brain (ESGIB). ESCMID guideline: Diagnosis and treatment of acute bacterial meningitis. Clin. Microbiol. Infect..

[32] Bosica S., Chiaverini A., De Angelis M.E., Petrini A., Averaimo D., Martino M., Rulli M., Saletti M.A., Cantelmi M.C., Ruggeri F. (2023). Severe *Streptococcus equi* Subspecies *zooepidemicus* Outbreak from Unpasteurized Dairy Product Consumption, Italy. Emerg. Infect. Dis..

